# TDR Technique for Estimating the Intensity of Evapotranspiration of Turfgrasses

**DOI:** 10.1155/2015/626545

**Published:** 2015-09-10

**Authors:** Grzegorz Janik, Karol Wolski, Anna Daniel, Małgorzata Albert, Wojciech Skierucha, Andrzej Wilczek, Paweł Szyszkowski, Amadeusz Walczak

**Affiliations:** ^1^Institute of Environmental Protection and Development, Wrocław University of Environmental and Life Sciences, Plac Grunwaldzki 24, 50-363 Wrocław, Poland; ^2^Department of Agroecosystems and Green Areas Management, Wrocław University of Environmental and Life Sciences, Plac Grunwaldzki 24, 50-363 Wrocław, Poland; ^3^Distance Learning Center, Wrocław University of Environmental and Life Sciences, Plac Grunwaldzki 24A, 50-365 Wrocław, Poland; ^4^Department of Metrology and Modelling of Agrophysical Processes, Institute of Agrophysics of the Polish Academy of Sciences, Doświadczalna 4, 20-290 Lublin, Poland; ^5^Institute of Landscape Architecture, Wrocław University of Environmental and Life Sciences, Plac Grunwaldzki 24A, 50-365 Wrocław, Poland

## Abstract

The paper presents a method for precise estimation of evapotranspiration of selected turfgrass species. The evapotranspiration functions, whose domains are only two relatively easy to measure parameters, were developed separately for each of the grass species. Those parameters are the temperature and the volumetric moisture of soil at the depth of 2.5 cm. Evapotranspiration has the character of a modified logistic function with empirical parameters. It assumes the form ETR(*θ* ^2.5 cm^, *T* ^2.5 cm^) = *A*/(1 + *B* · *e* ^−*C*·(*θ*^2.5 cm^ · *T*^2.5 cm^)^), where: ETR(*θ* ^2.5 cm^, *T* ^2.5 cm^) is evapotranspiration [mm·h^−1^], *θ* ^2.5 cm^ is volumetric moisture of soil at the depth of 2.5 cm [m^3^·m^−3^], *T* ^2.5 cm^ is soil temperature at the depth of 2.5 cm [°C], and *A, B*, and *C* are empirical coefficients calculated individually for each of the grass species [mm·h^1^], and [—], [(m^3^·m^−3^·°C)^−1^]. The values of evapotranspiration calculated on the basis of the presented function can be used as input data for the design of systems for the automatic control of irrigation systems ensuring optimum moisture conditions in the active layer of lawn swards.

## 1. Introduction

Limited water resources are a challenge in the maintenance of a suitable visual quality of lawns [1, 2]. Quantitative determination of evapotranspiration is of fundamental importance in the design of lawn irrigation systems [3, 4]. It depends on the kind and moisture of the soil, on the plant species, variety, and development phase of the grass, and on the atmospheric conditions [5–9]. One of the methods of estimating soil surface evaporation and water uptake by plant roots is the application of physical and mathematical empirical models [10–13]. Such models take into account a large number of input parameters. As an example, in the model developed by Fedes et al. [14] one should give the following: heat flux from the soil surface to the atmosphere, heat flux used for evaporation, heat flux from net radiation, latent heat of evaporation, vapour flux, and the incident heat flux received by the soil. In the empirical model one needs to additionally specify the root mass density and precisely define the physical properties of soil and for improved accuracy also determine the material functions of the soil [15]. In universal empirical models it is additionally required to analyse the input data from multiple synoptic stations. As an example, the estimation of ET0 by means of the Penman-Monteith model for the territory of Iran required the collection of data from as many as 181 synoptic stations [16–19]. Another method for estimation of ET0 is the application of energy budget methods in which parameters are determined by using the remote-sensing technique [20]. The intensity of evapotranspiration is also determined by the toilsome lysimetric research [21–23]. The objective of this study was the presentation of a method permitting precise calculation of evapotranspiration, with a short 1-hour time step, on the example of selected turfgrasses. The advantage of the method is that its application requires parameters that are relatively easy to measure—the temperature and moisture of the surface layer of soil. Since our considerations omitted the effect of the location of the lawn (built-up area, nonbuilt-up area, stadium, or green roof), calibration is required for every location. A similar approach, consisting in performing the calibration of Penman-Monteith (PM) formulae for a case when complete weather data are not available, was applied by Gao et al. [24]. In the study by Gao et al., the data required for the estimation of evapotranspiration (ET0) include temperature (*T*), relative humidity (RH), and sunshine duration (*n*). Yang et al. [25] applied an ETR estimation method based on meteorological data such as incoming solar radiation, air temperature, water vapour pressure, wind speed, and atmospheric pressure. The method presented here, after calibration, can also be applied to other plant species.

## 2. Materials and Methods

**Figure 1:**
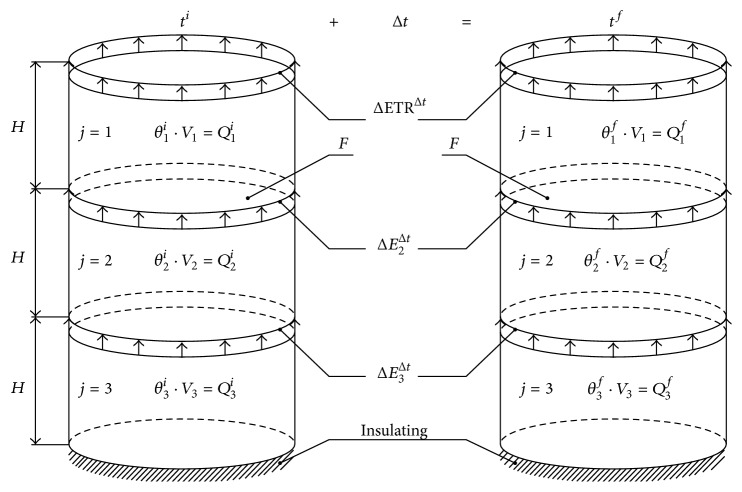
Dynamics of volumetric moisture as information on evapotranspiration; *θ*_*j*_^*i*^  (*θ*_*j*_^*f*^) is volumetric moisture in *j*th layer at the initial (final) moment [m^3^·m^−3^], *θ*_1,2,3_^*i*  (*f*)^ is volume of water in layers 1, 2, and 3 at the initial (final) moment [m^3^], ETR^Δ*t*^ is evapotranspiration [mm·h^−1^], *E*_2_^Δ*t*^  (*E*_3_^Δ*t*^) is unit intensity of water flux between layers 1 and 2 (2 and 3) [mm·h^−1^], *F* is surface area of soil column cross section [m^2^], *V*_*j*_ is volume of *j*th layer [m^3^], Δ*t* is time step [h], *t* ^*i*^  (*t* ^*f*^) is initial (final) moment, and *H* is soil layer height [m].

It was assumed that the evapotranspiration of selected turfgrass species can be calculated on the basis of a function whose domain is only two values: temperature and volumetric moisture of the surface layer of soil. The function can be determined on the basis of an experiment. The values that constitute its domain are relatively easy to measure, while the evapotranspiration can be calculated using the time-domain reflectometry (TDR), as illustrated in Figure 1.

**Figure 2:**
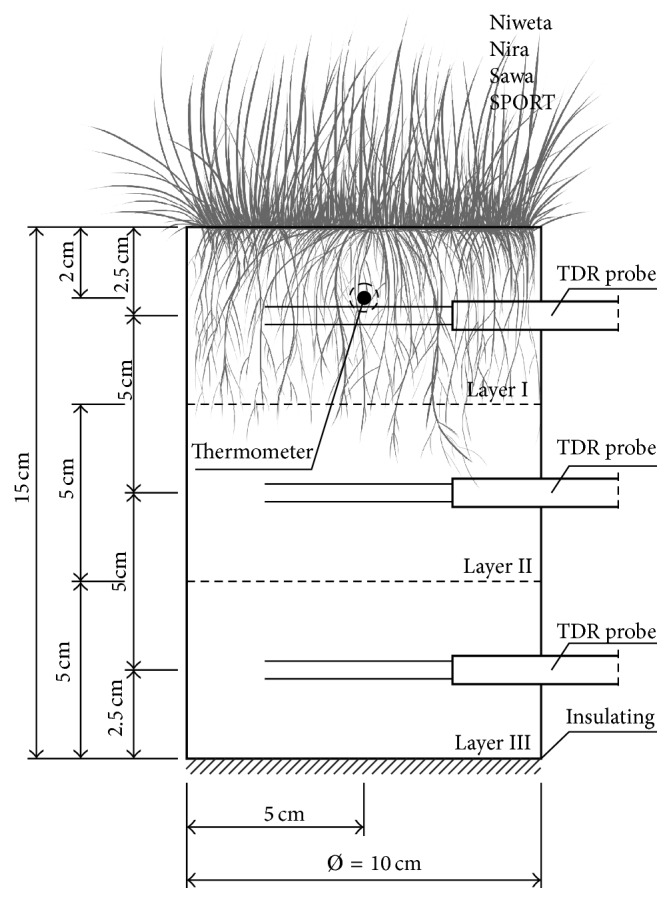
Schematic diagram of the experiment.

A soil column with plant root system was divided into 3 layers with identical volume *V*. The justification for the number of layers is given at the description of the Figure 2. During a nonrainfall period the water balance equation for the upper layer (*j* = 1) is as follows:

(1) $$\begin{array}{c} \theta_{1}^{i}\cdot V_{1}+E_{2}^{\Delta t}\cdot F\cdot \Delta t-{ETR}_{}^{\Delta t}\cdot F\cdot \Delta t=\theta_{1}^{f}\cdot V_{1}, \end{array}$$

where *θ*_1_^*i*^  (*θ*_1_^*f*^) is volumetric moisture in layer 1 at the initial (final) moment [m^3^·m^−3^], *V*_1_ is volume of layer 1 [m^3^], ETR^Δ*t*^ is evapotranspiration [m·h^−1^], *E*_2_^Δ*t*^ is intensity of water flux between layers 1 and 2  [m·h^−1^], Δ*t* is time step [h], and *F* is soil column cross-section surface area [m^2^].

In (1), the values of *θ*_1_^*i*^ and *θ*_1_^*f*^can be measured with high accuracy and freely selected with a small (even 1 minute) time step by means of the TDR technique. Values *V*, *F* result from the dimensions of the soil monolith. The value of time step Δ*t* is set individually for each experiment. The value of *E*_2_^Δ*t*^ is calculated in stages. First, we construct the balance equation for the bottom layer 3:

(2) $$\begin{array}{c} \theta_{3}^{i}\cdot V_{3}-E_{3}^{\Delta t}\cdot F\cdot \Delta t=\theta_{3}^{f}\cdot V_{3}, \end{array}$$

where *θ*_3_^*i*^  (*θ*_3_^*f*^) is volumetric moisture in layer 3 at the initial (final) moment [m^3^·m^−3^] and *E*_3_^Δ*t*^ is unit intensity of water flux between layers 2 and 3 [m·s^−1^], other symbols as in relation (1).

The only unknown of (2) is the value of *E*_3_^Δ*t*^. Then we construct the equation for layer 2:

(3) $$\begin{array}{c} \theta_{2}^{i}\cdot V_{2}+E_{3}^{\Delta t}\cdot F\cdot \Delta t-E_{2}^{\Delta t}\cdot F\cdot \Delta t=\theta_{2}^{f}\cdot V_{2}, \end{array}$$

where *θ*_2_^*i*^  (*θ*_2_^*f*^) is volumetric moisture in layer 2 at the initial (final) moment [m^3^·m^−3^], other symbols as in relations (1) and (2).

Finally, making use of relations (1), (2), and (3), we can calculate the loss of water from the whole volume of the monolith for time step Δ*t*:

(4) $$\begin{array}{c} {ETR}_{}^{\Delta t}=H\cdot {\Delta t}^{-1}\left(\;\theta_{1}^{i}-\theta_{1}^{f}+\theta_{2}^{i}-\theta_{2}^{f}+\theta_{3}^{i}-\theta_{3}^{f}\;\right), \end{array}$$

where *H* is height of the soil layer, other symbols as in relations (1), (2), and (3).

The experiment aimed at the determination of evapotranspiration using the method proposed above (4) was conducted in June 2013. Only an 11-day nonrainfall period, that is, from the 11th to the 21st of June, was taken into consideration for the analyses aimed at the construction of the evapotranspiration function. Cylindrical soil samples were covered with 4 different turfgrasses:* Poa pratensis* L. cult. Niweta,* Lolium perenne* L. cult. Nira,* Festuca rubra* L. cult. Sawa, and the grass mix SPORT (*Lolium perenne* L. (60%) cult. Nira, Niga;* Festuca rubra* L. (20%) cult. Sawa, Nimba;* Poa pratensis* L. (20%) cult. Alicja and Niweta). Further on in the text, they will be referred to as Niweta, Nira, Sawa, and grass mix SPORT, respectively. The grass species chosen—Niweta, Nira, and Sawa—are basic turfgrasses commonly used for sowing lawns and recreational areas. The grass mix SPORT is used primarily for football fields. The soil monoliths for the analyses were taken from an experiment setup with the split-plot method at the Agricultural Experimental Station Swojec in Wrocław (Poland) (E 17°08′22,56′′ N 51°06′59,04′′). That experiment was established on an alluvial soil developed on loamy sand, overlying light sand. The samples were taken so as to leave the soil structure undisturbed. Only the root systems were slightly damaged due to cutting on the edges of the monolith. The dimensions of the soil profiles are shown in Figure 2. They are the standard dimensions of turf samplers. The height of the soil column equals 15 cm. This is due to the fact that such is the depth of the vegetation horizon in the turf of football fields. Soil column diameter of *∅* = 10 cm results from the dimensions of the zone of sensitivity of the sensors used in the experiment. The soil profiles collected are homogeneous. The particle size distribution of the substrate conforms with the parameters required for the construction of the vegetation layer of sports objects (DIN 18035-4). As in the method described above, 3 layers were separated in the columns, with identical dimensions. The volumetric moisture was recorded at the centre points of the layers, using TDR probes type LP/ms, manufactured at the Institute of Agrophysics PAS in Lublin (Poland) [26]. In addition, an LP/*t* temperature sensor was installed at the central point of the top layer (at the depth of 2.5 cm). Based on a pilot study, it was found that, for a monolith height of 15 cm, the division of the column into 3 layers (3 TDR probes) is optimum for correct characterisation of moisture. Although increasing the number of layers (e.g., 5 TDR probes) might improve the accuracy of the water balance determined, at the same time it would cause greater disturbance of the water flow. It determines the depth of the sensors installation: 2.5 cm, 7.5 cm, and 12.5 cm. All measurements were made at the time step of Δ*t* = 1 h. In that way, a set of data was acquired to enable the calculation of the evapotranspiration for each measured temperature and moisture value of the top layer of soil. A schematic of probe distribution and of the experiment is presented in Figure 2.

## 3. Results

**Figure 3:**
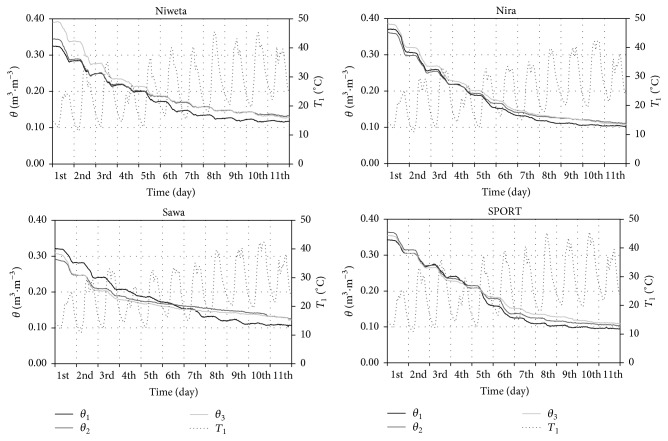
Dynamics of moisture in the period of 11–21.06.2013 for 4 grasses; *θ*_*j*_ is volumetric moisture in *j*th layer and *T*_1_ is temperature at the depth of 2.5 cm.

Figure 3 presents the measured data for each of the grasses—the dynamics of volumetric moisture in 3 soil layers and the dynamics of temperature in the top layers. The initial values of soil moisture (on the first day of the experiment) fall within the range from 0.3 m^3^·m^−3^ to nearly 0.4 m^3^·m^−3^.

**Figure 4:**
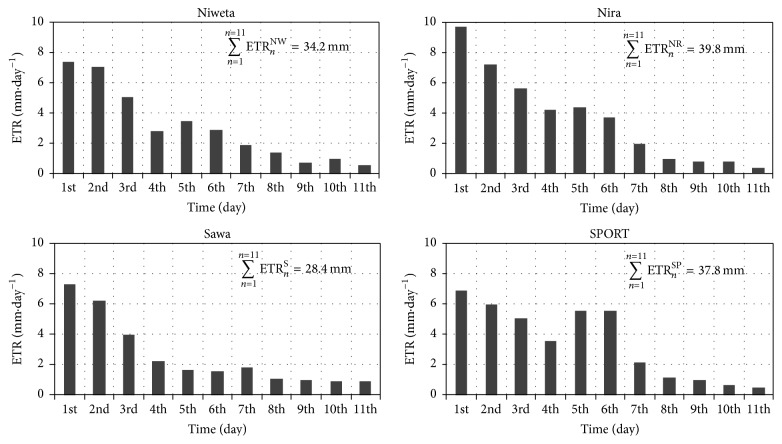
Dynamics of diurnal evapotranspiration in the period of 11–21.06.2013; ETR_*n*_ is evapotranspiration on *n*th day.

The maximum differences are found in layer 3. The initial volumetric moisture for Niweta is *θ*_3_^NW^ = 0.39 m^3^·m^−3^ and for cult. Sawa *θ*_3_^*S*^ = 0.31 m^3^·m^−3^. The differences may be caused by a lack of homogeneity of the soil profile, which occurs even in soils with the structure accepted as homogeneous. This regularity occurs for points situated even at small distances from each other [27]. In the period under analysis, the mean soil temperature at the depth of 2.5 cm, that is, in the turf horizon, was from 24.9°C for cult. Sawa to 26.0°C for cult. Niweta. The maximum temperature was very high. As an example, for cult. Niweta *T*_1_^NW^ = 45.5°C on the 18th of June (8th day). Significant drops of soil water content were observed, of course, during the daytime periods. For instance, for the lawn based on cult. Niweta, in layer 1 on the 13th of June (3rd day), the drop of moisture between the hours of 5:00 and 19:00 was 0.035 m^3^·m^−3^ and for cult. Nira 0.04 m^3^·m^−3^. It should be noted that the rate of the decrease was not uniform throughout the whole period. As an example, in layer 1 on the 12th of June (2nd day) the decrease for cv. Sawa was 0.041 m^3^·m^−3^·day^−1^ and on the 21st of June (11th day) only 0.001 m^3^·m^−3^·day^−1^. This was caused by the fact that on the 11th day the volumetric moisture of the soil column was lower than on the 2nd day. This shows that the diurnal evapotranspiration is the lower, the lower the volumetric moisture of the soil profile. That regularity occurs for each variety. The dynamics of diurnal evapotranspiration for each of the grasses is presented in Figure 4.

The values presented in the Figure were calculated from (4) with the time step of Δ*t* = 1 day. The adopted initial and final values (*θ*_*j*_^*i*^; *θ*_*j*_^*f*^) were the values from 0:00 hours on the successive days. The high mean diurnal temperature of the surface layer and, in the initial period, high water content were the cause of strong evapotranspiration. The maximum diurnal evapotranspiration, separately for each lawn, was observed on the 1st day of the experiment. It was 9.73 mm·day^−1^ for the lawn with cult. Nira, 7.37 mm·day^−1^ for Niweta, 7.28 mm·day^−1^ for Sawa, and 6.87 mm·day^−1^ for the grass mix SPORT. The analysis of those values indicates that under uniform moisture conditions of the soil profiles the maximum diurnal evapotranspiration for turfgrass Nira is higher than for the grass mix composed of several species of turfgrasses by 29.39% and 25.18% higher than for the lawn composed of cultivar Sawa. In the final period, for all of the turfgrasses the values of evapotranspiration are lower than 1 mm·day^−1^. The results for the grass SPORT are remarkable because of the significant decline of the ETR value. The observed effect was caused by the decrease of the maximum temperature on that day. For other grass species the decline in the value ETR also occurs, but it is not that sharp. The similarity of diurnal courses of evapotranspiration for all the turfgrasses is a proof that the application of the TDR technique permits correct calculation of the value of ETR with the time step Δ*t* = 1 day. Figure 4 also presents the total evapotranspiration for the period under analysis (∑_*n*=1_^*n*=11^ETR_*n*_). The highest value of total evapotranspiration (for the 11-day period) is observed for cult. Nira and amounts to 39.8 mm·day^−1^ and the lowest for cult. Sawa, at only 28.4 mm, that is, 28.64% less. This observation can be related with the information given in Figure 3 which shows that the mean moisture for the 3 layers is the highest for cultivar Nira and the lowest for cultivar Sawa. As in the case of evapotranspiration, the difference is approximately 25%. This can be a premise for the conclusion that the correlation between evapotranspiration and soil moisture is linear. At this stage this is a nonverified hypothesis which will be proven. In the next phase of the study an attempt was made at a more precise, that is, with a smaller time step Δ*t*, calculation of the value of ETR.

**Figure 5:**
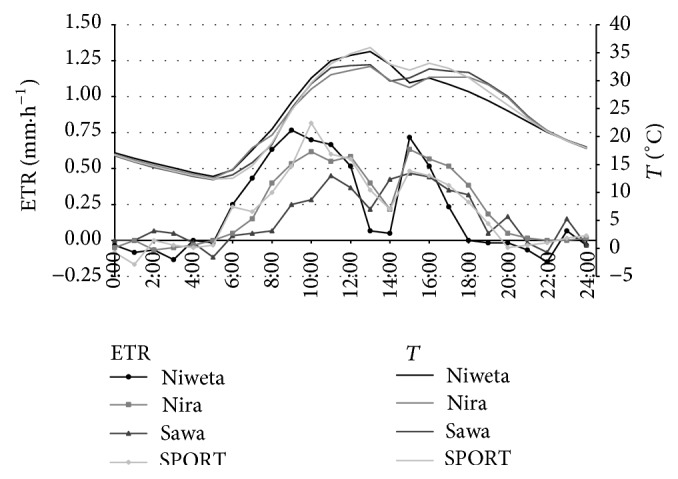
Diurnal dynamics of evapotranspiration and temperature for the four grasses on 13.06.2013 calculated with time step of Δ*t* = 1 h; ETR is evapotranspiration and *T* is soil temperature at the depth of 2.5 cm.

Figure 5 presents an example of the runs of evapotranspiration for each of the grasses during the period of one day, on the 13th of June (3rd day). These data were selected for graphic illustration as on that day there was a drop in insolation. Thanks to this, it was possible to observe a rapid change of evapotranspiration, which demonstrates the high sensitivity of the proposed method of measurement. The calculations were made with the time step of Δ*t* = 1 h using, as before, relation (4). During the hours from 0:00 to 5:00 and from 19:00 to 24:00, the values of ETR for each grass are zeroed. The occasional small negative values might have been caused by a lack of stability of the method for short time steps or an influx of water to the monoliths as a result of water infiltration from the atmosphere which occurs also during the nonrainfall periods [28]. During the day-time period, the variation of the values of ETR is as follows: in the morning, from 5:00 there is a systematic increase to the maximum value which is attained at 9:00 for Niweta, amounting to 0.77 mm·h^−1^, at 11:00 for Sawa, amounting to 0.45 mm·h^−1^, at 10:00 for Nira, amounting to 0.62 mm·h^−1^, and at 10:00 for the grass mix SPORT—0.82 mm·h^−1^. Then, for each of the grasses, a decrease of evapotranspiration takes place, until 13:00 for Sawa and till 14:00 for the remaining grasses. The minimum value of evapotranspiration during that period varies from 0.05 mm·h^−1^ for Niweta to 0.22 mm·h^−1^ for the remaining grasses. After that, in the afternoon hours slight increases take place in the values of ETR, up to values that amount to from 0.47 mm·h^−1^ for Sawa to 0.72 mm·h^−1^ for Niweta. The afternoon maxima are attained at 15:00, in spite of a slight drop in soil temperature at the depth of 2.5 cm at the time. This results from the fact that the maximum temperature on soil surface (h = 0) appears earlier than the maximum values at 16:00, for example, at 15:00. (The effect of differentiation of ETR values for the various grasses is related to the variation of moisture in the surface horizon of soil.) At 18:00 for Niweta and at 19:00 for the remaining grasses evapotranspiration ceases. The decrease of evapotranspiration in the afternoon hours is due to the fact that each day at that time the soil samples were in the shade. The shading, resulting from sample positioning, occurred from about 10:00 to approximately 13:00. It was also the cause of the disturbance in the course of the dynamics of temperature in the surface layers (Figure 5). Comparing the runs of evapotranspiration and temperature for the four grasses, we note that the maximum of evapotranspiration before noon takes place at 9:00 for Niweta, at 10:00 for Nira and the turfgrass mix, and at 11:00 for Sawa. Meanwhile, the maximum of temperature for each of the grasses occurs at 13:00. Moreover, the afternoon maximum of evapotranspiration for each grass occurs at 15:00 and of temperature at 16:00, except for Nira for which that maximum occurs at 17:00. This means that changes in evapotranspiration precede changes of temperature at the depth of 2.5 cm. That fact should be attributed to temperature changes at the depth of 2.5 cm being delayed in relation to soil temperature changes on its surface. Further on in the study, the effect of moisture and, at the same time, of the temperature of the surface layer of soil on the value of evapotranspiration was analysed.

**Figure 6:**
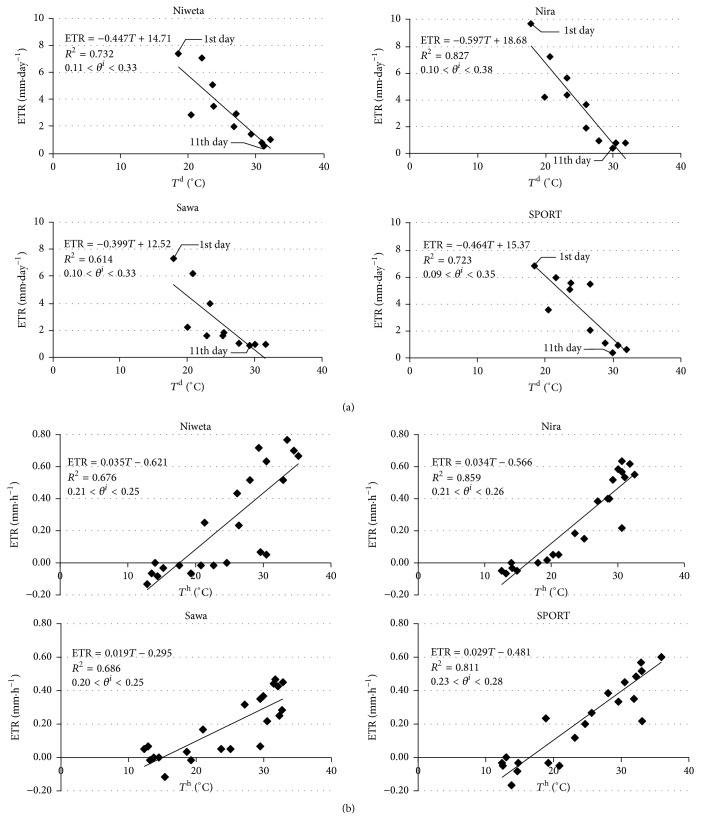
Relation of evapotranspiration to soil temperature at the depth of 2.5 cm. ETR is evapotranspiration, *T* ^d (h)^ is mean diurnal (hour) temperature of soil at the depth of 2.5 cm, and *θ*_*i*_^d^  (*θ*_*i*_^h^) is initial moisture for day (hour).

Figure 6(a) presents the relation of evapotranspiration, calculated from relation (4), with the time step Δ*t* = 1 day, with the mean diurnal soil temperature at the depth of 2.5 cm. The relation constructed in this way suggests that, with the increase in temperature, the diurnal values of evapotranspiration decrease which is untrue. In the experiment described, the decrease of the diurnal evapotranspiration is due to the fact that, with the passing of time, the volume of water decreases, and not to an increase of the mean temperature (Figure 3). For example, in the case of Niweta, when *θ*_*i*_^dNW^ on the 11th of  June (1st day) was 0.33 m^3^·m^−3^, ETR^NW^ = 7.37 mm·day^−1^. Meanwhile, on the 21st of June (11th day), when *θ*_*i*_^dNW^ was 0.12 m^3^·m^−3^, ETR^NW^ = 0.55 mm·day^−1^—likewise for the other grasses.  Figure 6(b) presents the same type of relationship, the difference being that the values of ETR were calculated with the time step of Δ*t* = 1 h. In this case, there is an increase of evapotranspiration with increase of the mean temperature for a given hour. This regularity results from the fact that the variation of evapotranspiration is caused only by changes of temperature, since moisture varies only slightly during 24 hours. For example, for* Poa pratensis* L. cult. Niweta on the 13th of June (3rd day) *θ* ^NW^ ∈ 〈0.21 m^3^ · m^−3^; 0.25 m^3^ · m^−3^〉. Further reduction of the time step Δ*t* causes that the values of  *R* ^2^ decrease. For instance, for Δ*t* = 15 min, *R* ^2^ = 0.47. This is due to the fact that for the short time steps the changes of moisture are smaller than the accuracy of measurement.

**Figure 7:**
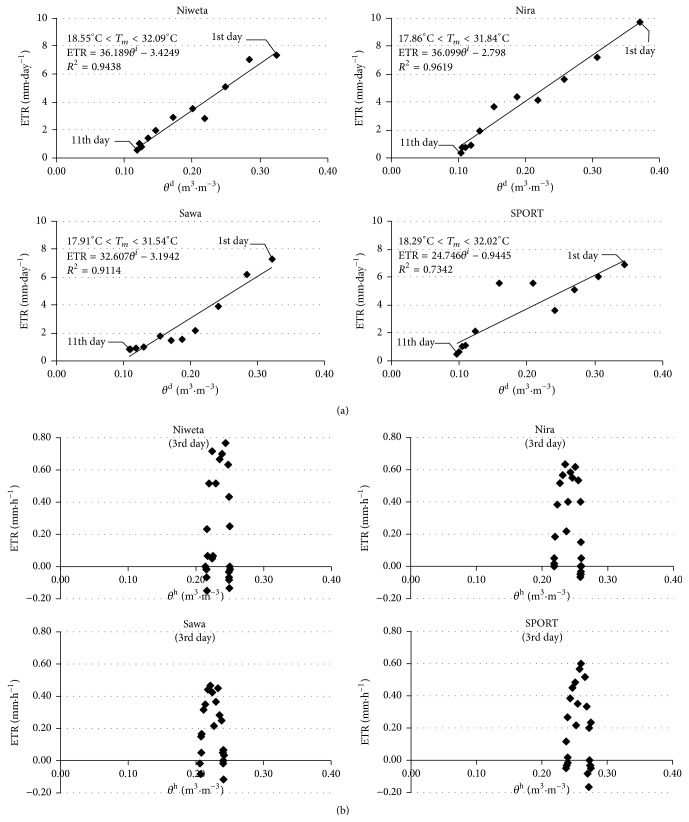
Relation of evapotranspiration to current moisture. ETR_d_ is diurnal evapotranspiration, ETR_h_ is hour evapotranspiration, *T*_*m*_ is mean temperature, and *θ* ^d (h)^ is mean diurnal (hour) moisture at the depth of 2.5 cm.

Figure 7 presents the relation of evapotranspiration to the mean diurnal (*θ* ^d^) or hour (*θ* ^h^) moisture of the surface layer of soil.  Figure 7(a) presents the value of ETR and the mean moisture calculated, as in the case of Figure 6, with the time step of Δ*t* = 1 day. For example, for Niweta the mean moisture of the surface layer of soil varied during the 11-day period from about 0.32 m^3^·m^−3^ to approximately 0.12 m^3^·m^−3^, and similar ranges were observed for the remaining grasses. Meanwhile, for all the grasses the diurnal values of evapotranspiration fell within the range from ~0.5 mm·day^−1^ when the soil moisture was ~0.1 m^3^·m^−3^ to as much as 10 mm·day^−1^, that is, when the soil moisture was 0.35 m^3^·m^−3^. The relation between diurnal evapotranspiration and soil moisture constructed in this way is proportional.  Figure 7(b) presents the relation of evapotranspiration, on the example of the 13th of June (3rd day), calculated with the time step Δ*t* = 1 h, with the mean soil moisture at a given hour. That relation is indeterminate, as the range of the changes of mean moisture during a 24-hour period is approximately 0.03 m^3^·m^−3^, and the variations in evapotranspiration are caused by diurnal temperature variation. Summarizing the analyses of Figures 6 and 7, we conclude that the construction of the relation of evapotranspiration to soil temperature at the depth of 2.5 cm and to its volumetric moisture cannot be conducted separately, with the same time step Δ*t*. Therefore, three-dimensional surfaces were constructed (further referred to as maps), described by 3 variables. In the horizontal plane, the coordinates are the volumetric moisture of soil and its temperature, measured at the depth of 2.5 cm, while the vertical dimension is evapotranspiration.

**Figure 8:**
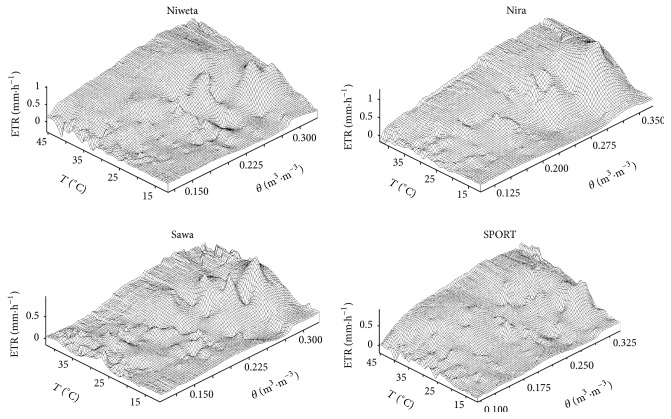
Maps of evapotranspiration for the turfgrasses. *θ* is volumetric moisture measured at the depth of 2.5 cm, *T* is temperature measured at the depth of 2.5 cm, and ETR is evapotranspiration.

Figure 8 presents the maps of evapotranspiration for the 4 grasses, plotted in the program Surfer. They are helpful in the choice of the type of the function approximating evapotranspiration. The shapes of the maps obtained indicate that the most adequate solution is to use the modified logistic function for the description. The domain of a function is the volumetric moisture and temperature at the depth of 2.5 cm (*θ* ^2.5 cm^, *T* ^2.5 cm^). It assumes the following form:

(5) $$\begin{array}{c} ETR\left(\;\theta^{2.5\;cm},T^{2.5\;cm}\;\right)=\frac{A}{1+{B\cdot e}^{-C\cdot \left(\theta^{2.5\;cm}\cdot T^{2.5\;cm}\right)}}, \end{array}$$

where ETR(*θ* ^2.5 cm^, *T* ^2.5 cm^) is evapotranspiration [mm·h^−1^], *θ* ^2.5 cm^ is volumetric moisture at the depth of 2.5 cm [m^3^·m^−3^], *T* ^2.5 cm^ is temperature at the depth of 2.5 cm [°C], and *A*, *B*, and *C* are empirical coefficients calculated individually for each turfgrass species [mm·h^1^], [—], and [(m^3^·m^−3^·°C)^−1^].

The values of parameters *A*, *B*, and *C* were chosen so that the sum of the square differences (*R* ^2^) calculated for the values of ETR by means of relations (4) and (5) was the smallest. Microsoft Office Excel (Solver) was used for this purpose. The values of *R* ^2^ were calculated on the basis of the relation:

(6) $$\begin{array}{c} R^{2}=\frac{1}{n}\overset{n}{\underset{i=1}{\sum }}{\left[\;{ETR}_{}^{\Delta t}-ETR\left(\;\theta^{2.5\;cm},T^{2.5\;cm}\;\right)\;\right]}^{2}, \end{array}$$

where *R* ^2^ is mean square difference between the values of ETR^Δ*t*^ and ETR(*θ* ^2.5 cm^, *T* ^2.5 cm^) [mm·h^−1^] and *n* is number of pairs compared, other symbols as in relations (4) and (5).

In addition, to estimate the similarity of ETR^Δ*t*^ and ETR(*θ* ^2.5 cm^, *T* ^2.5 cm^), the mean module of the differences was calculated from the following formula:

(7) $$\begin{array}{c} K=\frac{1}{n}\overset{n}{\underset{i=1}{\sum }}\left|\;{ETR}_{}^{\Delta t}-ETR\left(\;\theta ,T\;\right)\;\right|, \end{array}$$

where *K* is mean module of the differences between the values of ETR^Δ*t*^ and ETR(*θ*, *T*) [mm·h^−1^], other symbols as in relation (6).

The values of parameters *A*, *B*, and *C* and the measures of fit *R* ^2^ and *K* are presented in Table 1.

Table 1 also presents the values of the empirical parameters *A*, *B*, and *C* which guarantee the obtainment of the minimum values of *R* ^2^ and *K*. Parameter *A* determines the range of values of ETR [ETR^Δ*t*^ ∈ (0, *A*)]. Parameters *B* and *C* determine the rate of initiation of the process of function saturation, that is, its shape. The range of variation of the mean square differences and the mean modules of differences indicate the quality of approximation. The values of *R* ^2^ vary from 0.006 for the grass mix* SPORT* to 0.011 for var.* Niweta*. This indicates very good fit of the values calculated on the basis of formula (4) to the function defined by formula (5).

The relations developed in the above manner were subjected to validation using independent research material. Based on the measurements of volumetric moisture in all soil layers, the values of ETR^Δ*t*^ were calculated from formula (4). Next, using measurements of volumetric moisture and temperature at the depth of 2.5 cm and the models developed, the values of ETR(*θ* ^2.5 cm^, *T* ^2.5 cm^) were calculated from formula (5). Model accuracy was determined using measures *R*_*w*_^2^ and *K*_*w*_, calculated analogously as in formulae (6) and (7).

Table 2 shows the measures of fit of the model which verifies its usability. The measure of the good fit of the model *R*_*w*_^2^ varies from 0.025 [mm·h^−1^]^2^ for grass mix* SPORT* to 0.056 [mm·h^−1^]^2^ for cultivar* Nira*. Meanwhile, measure *K*_*w*_ varies from 0.10 [mm·h^−1^] for grass mix* SPORT* to 0.13 [mm·h^−1^] for cultivars* Sawa* and* Niweta*. The results obtained indicate the correctness of the approximation model adopted which may constitute a basis for the method of precise estimation of the process of evapotranspiration.

## 4. Conclusions

(1) Evapotranspiration functions were developed for selected species of turfgrasses. The arguments of the functions are volumetric moisture and temperature of soil at the depth of 2.5 cm. This permits precise simulation of the value of ETR with a short, even as short as one-hour, time step.
(2) Evapotranspiration has the character of a modified logistic function with empirical parameters. The function has been given in (5)
  (8) $$\begin{array}{c} ETR\left(\;\theta^{2.5\;cm},T^{2.5\;cm}\;\right)=\frac{A}{1+{B\cdot e}^{-C\cdot \left(\theta^{2.5\;cm}\cdot T^{2.5\;cm}\right)}}, \end{array}$$
 where ETR(*θ* ^2.5 cm^, *T* ^2.5 cm^) is evapotranspiration [mm·h^−1^], *θ* ^2.5 cm^ is volumetric moisture at the depth of 2.5 cm [m^3^·m^−3^], *T* ^2.5 cm^ is temperature at the depth of 2.5 cm, and *A*, *B*, and *C* are empirical coefficients calculated individually for each of the grass species [mm·h^1^], [—], and [(m^3^·m^−3^·°C)^−1^].
(3) The values of evapotranspiration calculated on the basis of the presented function can be used as input data for the design of systems of automatic control of irrigation systems ensuring optimum moisture conditions in the active layer of lawn swards.
(4) The method presented here permits the selection of grass species for the purpose of limitation of evapotranspiration. For example, the maximum diurnal evapotranspiration for a monoculture lawn composed of cultivar Nira is higher than for a turf composed of several species of turfgrasses by 29.39% and 25.18% higher than that of a lawn composed of cultivar Sawa.

## Figures and Tables

**Table 1:** Parameters *A*, *B*, and *C* and measures of fit *R* ^2^ and *K*. *R* ^2^ is calculated from relation (6) and *K* is calculated from relation (7).

Grass	Parameter *A* [mm·h^−1^]	Parameter *B* [—]	Parameter *C* [(m^3^·m^−3^·°C)^−1^]	*R* ^2^ [mm·h^−1^]^2^	*K* [mm·h^−1^]
*Niweta*	0.91	89.29	0.69	0.011	0.10
*Nira*	0.95	190.50	0.66	0.007	0.04
*Sawa*	0.91	185.29	0.82	0.009	0.08
*SPORT*	0.90	122.31	0.62	0.006	0.06

**Table 2:** Measures of good fit of the model.

Grass	*R*_*w*_^2^ [mm·h^−1^]^2^	*K*_*w*_ [mm·h^−1^]
*Niweta*	0.034	0.13
*Nira*	0.056	0.12
*Sawa*	0.030	0.13
*SPORT*	0.025	0.10

## References

[1] Bushman B. S., Waldron B. L., Robins J. G., Bhattarai K., Johnson P. G. (2012). Summer percent green cover among Kentucky bluegrass cultivars, accessions, and other *Poa* species managed under deficit irrigation. *Crop Science*.

[2] Merewitz E., Meyer W., Bonos S. A., Huang B. (2010). Drought stress responses and recovery of Texas × Kentucky hybrids and kentucky bluegrass genotypes in temperate climate conditions. *Agronomy Journal*.

[3] Carrow R. N. (1995). Drought resistance aspects of turfgrasses in the southeast: evapotranspiration and crop coefficients. *Crop Science*.

[4] Ebdon J. S., Kopp K. L. (2004). Relationships between water use efficiency, carbon isotope discrimination, and turf performance in genotypes of Kentucky bluegrass during drought. *Crop Science*.

[5] Bousselot J. M., Klett J. E., Koski R. D. (2011). Moisture content of extensive green roof substrate and growth response of 15 temperate plant species during dry down. *HortScience*.

[6] Bremer D., Lewis J., Keeley S., Fry J. (2012). Effects of wilt-based irrigation on visual quality and seasonal water applications on 30 bluegrasses in the transition zone. *USGA Turfgrass and Environmental Research Online*.

[7] Domenghini J., Bremer D. J., Fry J. D., Davis G. L. (2013). Evapotranspiration and performance among turfgrass and 22 ornamental landscape species in response to irrigation deficit. *International Turfgrass Society Research Journal*.

[8] Lewis J. D., Bremer D. J., Keeley S. J., Fry J. D. (2012). Wilt-based irrigation in *Kentucky bluegrass*: effects on visual quality and irrigation amounts among cultivars. *Crop Science*.

[9] Wang P., Yamanaka T. (2014). Application of a two-source model for partitioning evapotranspiration and assessing its controls in temperate grasslands in central Japan. *Ecohydrology*.

[10] Kochendorfer J. P., Ramírez J. A. (2010). Modeling the monthly mean soil-water balance with a statistical-dynamical ecohydrology model as coupled to a two-component canopy model. *Hydrology and Earth System Sciences*.

[11] Loheide S. P. (2008). A method for estimating subdaily evapotranspiration of shallow groundwater using diurnal water table fluctuations. *Ecohydrology*.

[12] Morari F., Giardini L. (2001). Estimating evapotranspiration in the Padova botanical garden. *Irrigation Science*.

[13] Suleiman A. A., Hoogenboom G. (2007). Comparison of priestley-taylor and FAO-56 penman-monteith for daily reference evapotranspiration estimation in Georgia. *Journal of Irrigation and Drainage Engineering*.

[14] Fedes R., Kowalik P., Zaradny H. (1978). *Simulation of Field Water Use and Crop Yield*.

[15] Soylu M. E., Kucharik C. J., Loheide S. P. (2014). Influence of groundwater on plant water use and productivity: development of an integrated ecosystem—variably saturated soil water flow model. *Agricultural and Forest Meteorology*.

[16] Valipour M. (2015). Temperature analysis of reference evapotranspiration models. *Meteorological Applications*.

[17] Valipour M. (2014). Use of average data of 181 synoptic stations for estimation of reference crop evapotranspiration by temperature-based methods. *Water Resources Management*.

[18] Valipour M. (2015). Comparative evaluation of radiation-based methods for estimation of potential evapotranspiration. *Journal of Hydrologic Engineering*.

[19] Valipour M. (2014). Importance of solar radiation, temperature, relative humidity, and wind speed for calculation of reference evapotranspiration. *Archives of Agronomy and Soil Science*.

[20] Rahimia S., Sefidkouhia M. A. G., Raeini-Sarjaza M., Valipourb M. (2015). Estimation of actual evapotranspiration by using MODIS images (a case study: Tajan catchment). *Archives of Agronomy and Soil Science*.

[21] Bakhtiari B., Ghahreman N., Liaghat A. M., Hoogenboom G. (2011). Evaluation of reference evapotranspiration models for a semiarid environment using lysimeter measurements. *Journal of Agricultural Science and Technology*.

[22] Chávez J. L., Gowda P. H., Howell T. A., Garcia L. A., Copeland K. S., Neale C. M. U. (2012). ET mapping with high-resolution airborne remote sensing data in an advective semiarid environment. *Journal of Irrigation and Drainage Engineering*.

[23] Kowalik P. J. (2006). Drainage and capillary rise components in water balance of alluvial soils. *Agricultural Water Management*.

[24] Gao X., Peng S., Xu J., Yang S., Wang W. (2015). Proper methods and its calibration for estimating reference evapotranspiration using limited climatic data in Southwestern China. *Archives of Agronomy and Soil Science*.

[25] Yang Y., Su H., Zhang R., Wu J., Qi J. (2013). A new evapotranspiration model accounting for advection and its validation during SMEX02. *Advances in Meteorology*.

[26] Skierucha W., Wilczek A., Szypłowska A., Sławiński C., Lamorski K. (2012). A TDR-based soil moisture monitoring system with simultaneous measurement of soil temperature and electrical conductivity. *Sensors*.

[27] Janik G. (2008). Spatial variability of soil moisture as information on variability of selected physical properties of soil. *International Agrophysics*.

[28] Janik G., Skierucha W., Błaś M. (2014). TDR technique for estimating the intensity of effective non rainfall. *International Agrophysics*.

